# Biases underlying species detection using fluorescent amplified-fragment length polymorphisms yielded from roots

**DOI:** 10.1186/s13007-015-0079-1

**Published:** 2015-06-26

**Authors:** Justine Karst, Pak Chow, Simon M Landhäusser

**Affiliations:** Department of Renewable Resources, University of Alberta, 442 Earth Sciences Building, Edmonton, AB T6G 2E3 Canada

**Keywords:** *trn*T-*trn*L intergenic spacer, *trn*L intron, *trn*L-*trn*F intergenic spacer, AFLP, *Populus tremuloides* Michx., *Bromus inermis* Leyss., Mixed template, False-negatives, belowground diversity, Community

## Abstract

**Background:**

Roots of different plant species are typically morphologically indistinguishable. Of the DNA-based techniques, fluorescent amplified-fragment length polymorphisms (FAFLPs) are considered reliable, high throughput, inexpensive methods to identify roots from mixed species samples. False-negatives, however, are not uncommon and their underlying causes are poorly understood. We investigated several sources of potential biases originating in DNA extraction and amplification. Specifically, we examined the effects of sample storage, tissue, and species on DNA yield and purity, and the effects of DNA concentration and fragment size on amplification of three non-coding chloroplast regions (*trn*T-*trn*L intergenic spacer, *trn*L intron, and *trn*L-*trn*F intergenic spacer).

**Results:**

We found that sample condition, tissue and species all affected DNA yield. A single freeze–thaw reduces DNA yield, DNA yield is less for roots than shoots, and species vary in the amount of DNA yielded from extractions. The effects of template DNA concentration, species identity, and their interaction on amplicon yield differed across the three chloroplast regions tested. We found that the effect of species identity on amplicon production was generally more pronounced than that of DNA concentration. Though these factors influenced DNA yield, they likely do not have a pronounced effect on detection success of fragments and only underscore the restriction on the use of FAFLPs for measuring species presence rather than their abundance. However, for two of the regions tested—the *trn*T-*trn*L intergenic spacer and the *trn*L intron—size-based fragment competition occurred and the likelihood of detection was higher for smaller than larger fragments. This result reveals a methodological bias when using FAFLPs.

**Conclusions:**

To avoid potential bias with the use of FAFLPs, we recommend users check for the disproportionate absence of species detected belowground versus aboveground as a function of fragment size, and explore other regions, aside from the *trn*T-*trn*L intergenic spacer and *trn*L intron, for amplification.

**Electronic supplementary material:**

The online version of this article (doi:10.1186/s13007-015-0079-1) contains supplementary material, which is available to authorized users.

## Background

The analysis of plant communities in relation to abiotic and biotic factors has mostly emerged from studying aboveground responses. Roots, however, are the primary organ of water and nutrient acquisition, account for a major portion of primary production, and may mediate aboveground coexistence and diversity from their interactions with other plants and organisms occurring in soils [1, 2]. How roots are organized belowground compared with the spatial organization of aboveground organs has only recently been investigated [3–6]. Our understanding of ecological factors governing root placement, foraging and function lags far behind that of aboveground plant organs owing to practical difficulties with sampling and identifying roots to the level of species. Roots of different species are typically morphologically indistinguishable. A variety of methods (e.g., morphological, chemical, spectroscopic and fluorescent) exist to identify roots, however, DNA-based molecular markers hold the most promise because they do not vary depending on abiotic and biotic conditions [7]. Specifically, molecular identification using fluorescent amplified-fragment length polymorphisms (FAFLPs) is considered a reliable and inexpensive method to determine species identity of roots in soils [8, 9]. This method has been advocated for use in species-rich plant communities where high sample throughput is required for analysis, and multiple species co-occur within a sample (i.e., mixed template). Sequence-based markers are used to differentiate species using size differences in fluorescently labelled PCR amplicons. Size profiles derived from roots are then compared to those developed from leaves of identified species occurring within a species pool defined by the user. For instance, using two non-coding regions of cpDNA in roots, 80% of 95 plants in a fescue grassland were identified [10]. Despite this success, often species recorded aboveground are not detected belowground (i.e., false-negatives), and the reasons for this are poorly understood [11].

Aside from actual differences between above and belowground plant richness [5, 12], the molecular methods used for FAFLPs may give rise to false-negatives. In particular, false-negatives may originate in DNA extraction, amplification, and quantification of amplified fragments. Of course, differences in the abundance of roots occurring in soils affect the amount of root DNA, and this relationship has been investigated towards developing quantitative real-time PCR methods and detection thresholds [13, 14]. Regarding DNA extraction, chemicals present in plant material vary by age, tissue and species, and these differences in underlying chemistry can affect DNA yields [11, 15–17]. In addition to chemistry, the ‘freshness’ of material and differences in sample storage can also influence DNA extraction. Though sample condition has been tested on roots of herbaceous species [18], woody tree roots often require mechanical treatment to disrupt cell walls, thus they may differ from herbaceous species in their sensitivity to extraction and storage conditions. Of the limited research investigating how plant material and DNA concentration affects DNA yield from roots, most of it has been with species from a particular ecosystem (e.g. grassland or forest) and not for use in FAFLPs. With global change and shifts in human land-use, many ecosystems are in a state of conversion [19], consequently, comparisons between life forms (e.g. trees versus grasses) are increasingly necessary. While other molecular methods are available to identify mixed template, in particular, next generation sequencing, these methods are expensive compared with FAFLPs, informatics-intensive, and not necessarily the desired tool when sample numbers are high as is the usual case for community studies.

Following DNA extraction, subsequent biases can arise in amplification. For example, differences across species and in root diameter influenced the recovery efficiency of species present in clone libraries [20]. Several studies have reported that the complexity and intensity of fragments decreases with DNA concentration, indicating that fragments are not randomly distributed at low DNA concentrations [21, 22]. One explanation for this finding is that high species richness of DNA template may induce competition among primers, thus selecting for short fragments. Taggart et al. [10] tested for primer competition using trials of mixed samples containing between 4 and 16 grassland species. In that study, the increase in false-negatives in samples of higher species diversity was attributed to a higher probability of ‘difficult’ species present in samples of higher diversity rather than a result of primer competition. However, neither fragment size nor template concentration was considered in that study. False-negatives may also be present post-amplification as fluorescently labeled amplicons are injected into capillaries; shorter fragments have greater mobility into the capillaries and therefore may be more detectable than longer fragments. This bias was tested by combining amplicons yielded from seven boreal tree species (and thus of different sizes) post-amplification in trials of one, two, four, and six species; no evidence of fragment competition was discerned [8].

In short, plant species vary in abundance, either through the amount of roots present in a soil sample or through the DNA they yield. Users employ different strategies to conserve samples, potentially affecting DNA quality and quantity. Species vary in sizes of target DNA, which may be preferentially amplified based on size. Collectively, no single study has assessed these sources of bias with use of FAFLPs. We focussed our work on two stages: DNA extraction and amplification. To identify biases arising with DNA extraction, we tested the effects of species, tissue and sample storage on DNA yields and purity with species from forests prone to invasion by grassland species. Using these same species combined with others from a similar ecosystem, we tested biases arising with amplification. Specifically, we tested how species and DNA concentration affected amplicon yield, and as a result, detection thresholds. We also tested how DNA concentration and fragment size affected fragment presence by creating known mixtures of plant species and manipulating the abundance of a given component of the mixture. Taken together, this study highlights methodological issues affecting false-negatives in the species identification of roots using FAFLPs.

## Results

### DNA extraction: testing for differences in DNA yield and purity by tissue, species and sample condition

**Figure 1:**
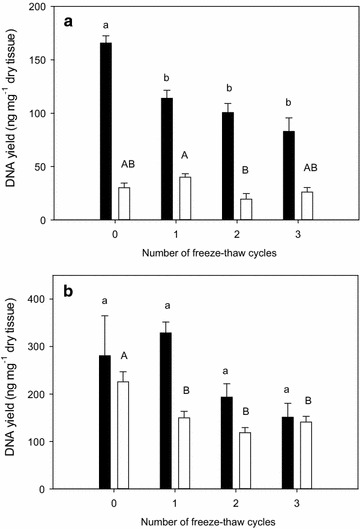
Influence of tissue and sample condition on DNA yield. For a tissue type (leaves: *black bars*, roots: *white bars*), means (± SE) labeled with *different letters* are significantly different at *P* < 0.05 based on Bonferroni post hoc tests. N = 5–6 for **a** *Populus tremuloides* and **b** N = 6 for *Bromis inermis.*

Species, tissue, and sample condition all significantly affected DNA yield (Additional file 1: Table S3). Overall DNA yield was higher for *Bromus inermis* (199 ± 15 ng mg^−1^ dry tissue) than *Populus tremuloides* (74 ± 7 ng mg^−1^ dry tissue). Across both species, roots yielded less DNA than leaves (Figure 1). The difference in yield between leaves and roots was proportionally less for *Bromus inermis* than *Populus tremuloides*. DNA yield of roots was 66% and 25% that of leaves for *Bromus inermis* and *Populus tremuloides*, respectively. Thawing frozen samples generally decreased DNA yields; however, the number of freeze–thaw cycles did not have a pronounced effect on DNA quantity (Figure 1). Regardless of species, there was no effect of sample condition on DNA purity measured as A_260_/A_280_ absorbance ratios (Additional file 1: Table S4). Mean A_260_/A_280_ absorbance ratio was highest for DNA extracted from roots of *Populus tremuloides* (1.86 ± 0.026) compared with that for leaves (1.74 ± 0.0121). Absorbance ratios were similar for DNA extracted from leaves and roots of *Bromus inermis* (1.81 ± 0.020 and 1.83 ± 0.007, respectively).

### Amplification: testing the effect of species and DNA concentration on amplicon yield

Between root and leaf tissue, fragment sizes varied within one base pair, i.e., fragments yielded from root and leaf tissue of a single individual were effectively sized the same. Across the three regions, fragments differed in size between *Populus tremuloides* and *Bromus inermis* (Table 1), and some intraspecific variation was present in fragments of the *trn*L intron for *Populus tremuloides*. We found that for the *trn*L-*trn*F intergenic spacer, individuals of *Bromus inermis* expressed two fragments (Table 1). DNA extracted from leaves yielded fragments sized 394–395 base pairs (bp) and occurred eight times less than fragments sized 443 bp [*t*(10) = −6.28, P < 0.001] However, DNA extracted from roots yielded fragments sized 394–395 bp and occurred in equal abundance as fragments sized 443 bp [*t*(10) = −0.46, P = 0.66]. Amplification of DNA extracted from frozen-thawed samples produced amplicons of the same fragment lengths as those from fresh samples (data not shown) for all three chloroplast non-coding regions.

**Table 1:** Fragment sizes of three regions of chloroplast DNA

Species	Region		
	*trn*T-*trn*L intergenic spacer	*trn*L intron	*trn*L-*trn*F intergenic spacer
*Populus tremuloides*	529–530	685–706	391–393
*Bromus inermis*	377, 664	649	(394–395), 443
*Picea glauca*	466, 475	559–560^a^	460^a^
*Melilotus officinalis*	1,154	319	205, 216^a^
*Sonchus arvensis*	635, 636^a^	507	417^a^
*Chamerion angustifolium*	n/a	603	365
*Trifolium hybridum*	n/a	585, 615^a^	209
*Rubus idaeus*	n/a	556–567^a^	476

Fragment size was measured in base pairs for plant species (n = 6) common in western Canada.

n/a unsuccessful amplifications.

^a^Unpublished values determined by the authors in previous trials using same conditions of extraction, amplification and fragment analysis.

**Figure 2:**
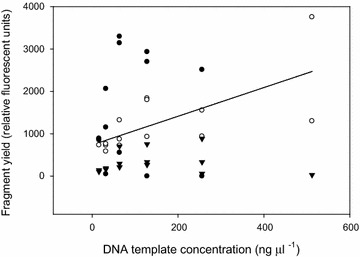
Relationship between DNA template concentration and fragment yield of *trn*T-*trn*L intergenic spacer. Three species are shown: *Populus tremuloides* (*closed circles*; n.s.), *Melilotus officinalis* (*triangles*; n.s.) and *Bromus inermis* (*open circles*; y = 761 + 3.6x, *R* ^2^ = 0.48).

The effects of DNA concentration, species, and their interaction on amplicon yield differed by region. Amplicon yield of the *trn*T-*trn*L intergenic spacer was affected by the interaction between DNA concentration and species (Additional file 1: Table S5). Amplicon yield of *Bromus inermis* increased with DNA concentration, whereas *Melilotus officinalis* and *Populus tremuloides* did not (Figure 2). Species and DNA concentration independently affected amplicon yield of the *trn*L intron (Additional file 1: Table S6). There was a weak, positive relationship between DNA concentration and amplicon yield (R^2^ < 0.1; data not shown). There was an order of magnitude difference among mean amplicon yield across species; *Melilotus officinalis* had the highest yield (62,672 ± 329 rfu), followed by *Bromus inermis* (11,987 ± 957 rfu), and *Populus tremuloides* (2,739 ± 361 rfu). Similar to amplicons of *trn*L intron, species identity also had the most pronounced effect on amplicon yield of the *trn*L-*trn*F intergenic spacer (Additional file 1: Table S7). *Melilotus officinalis* and *Populus tremuloides* had similar yields, 19,774 ± 2,429 rfu and 18,857 ± 2,045 rfu, respectively. *Bromus inermis* had the least (5,716 ± 810 rfu).

### Amplification: testing the effect of DNA concentration and fragment size on fragment detection

Across all trials, there were no false-positives, and only one sample failed to give fragment sizing results. For all three chloroplast regions, fragment sizes differed across known plant species (Table 1).

Regarding species pools assembled with DNA extracted from leaves, for the *trn*T-*trn*L intergenic spacer, *Picea glauca* (466 and 475 bp) and *Melilotus officinalis* (1,154 bp) were not detected regardless of DNA concentration (Table 2). Both *Bromus inermis* (664 bp) and *Populus tremuloides* (530 bp) were detected across the concentration gradient (2.73–22.73 ng μL^−1^) aside from the smaller fragment (377 bp) of *Bromus inermis* absent at the lowest concentration. For the *trn*L intron, both *Melilotus officinalis* (319 bp) and *Sonchus arvensis* (508 bp) were present across the concentration gradient (0.045–0.45 ng μL^−1^), and both *Chamerion angustifolium* (603 bp) and *Populus tremuloides* (706 bp) were absent (Table 3). For the *trn*L-*trn*F intergenic spacer, both *Trifolium hybridum* (209 bp) and *Chamerion angustifolium* (365 bp) were present across the concentration gradient, and the smaller fragment (394 bp) of *Bromus inermis* was absent. *Populus tremuloides* (476 bp) and the larger fragment of *Bromus inermis* (443 bp) were absent at the lowest concentration (0.045 ng μL^−1^), but present otherwise (Table 2).

**Table 2:** Detection success of amplicons yielded from multi-species mixtures

cpDNA	Fragment size (bp)	Species	Detection rate		
			2.73 ng μL^−1^ DNA	12.5 ng μL^−1^ DNA	22.73 ng μL^−1^ DNA
*trn*T-*trn*L intergenic spacer	377	*Bromus inermis* ^a^	0/3	3/3	3/3
*trn*T-*trn*L intergenic spacer	466	*Picea glauca* ^a^	0/3	0/3	0/3
*trn*T-*trn*L intergenic spacer	475	*Picea glauca* ^a^	0/3	0/3	0/3
*trn*T-*trn*L intergenic spacer	530	*Populus tremuloides*	3/3	3/3	3/3
*trn*T-*trn*L intergenic spacer	664	*Bromus inermis* ^a^	3/3	3/3	3/3
*trn*T-*trn*L intergenic spacer	1,154	*Melilotus officinalis*	0/3	0/3	0/3

cpDNA	Fragment size (bp)	Species	Detection rate		
			0.045 ng μL^−1^ DNA	0.25 ng μL^−1^ DNA	0.45 ng μL^−1^ DNA
*trn*L intron	319	*Melilotus officinalis*	3/3	3/3	3/3
*trn*L intron	508	*Sonchus arvensis*	3/3	3/3	3/3
*trn*L intron	603	*Chamerion angustifolium*	0/3	0/3	0/3
*trn*L intron	706	*Populus tremuloides*	0/3	0/3	0/3
*trn*L-*trn*F intergenic spacer	209	*Trifolium hybridum*	3/3	3/3	3/3
*trn*L-*trn*F intergenic spacer	365	*Chamerion angustifolium*	3/3	3/3	3/3
*trn*L-*trn*F intergenic spacer	394	*Bromus inermis* ^a^	0/3	0/3	0/3
*trn*L-*trn*F intergenic spacer	443	*Bromus inermis* ^a^	0/3	3/3	3/3
*trn*L-*trn*F intergenic spacer	476	*Populus tremuloides*	0/3	3/3	3/3

DNA was extracted from leaves of identified species and mixed in varied proportions with other known plant species. Numerator is number of successful detections; denominator is number of trials.

^a^This species yielded fragments of different sizes.

**Table 3:** Detection success of amplicons yielded from species mixtures containing identified and undetermined plant species

cpDNA	Fragment size (bp)	Species	Soil source^a^	Detection rate		
				4.5 ng μL^−1^ DNA	25 ng μL^−1^ DNA	45.5 ng μL^−1^ DNA
*trn*T-*trn*L intergenic spacer	531	Undetermined	1, 2	0/6	1/6	5/6
*trn*T-*trn*L intergenic spacer	637	Undetermined	1	0/3	2/3	3/3
*trn*T-*trn*L intergenic spacer	679	Undetermined	2	0/3	6/6	3/3
*trn*T-*trn*L intergenic spacer	881	Undetermined	1, 2	0/6	6/6	6/6
*trn*T-*trn*L intergenic spacer	1,154	*Melilotus officinalis*	n/a	0/6	6/6	6/6

cpDNA	Fragment size (bp)	Species	Soil source^a^	Detection rate		
				0.09 ngμL^−1^ DNA	0.5 ng μL^−1^ DNA	0.9 ng μL^−1^ DNA
*trn*L intron	320	Undetermined	1	0/3	2/2	3/3
*trn*L intron	490	Undetermined	1	3/3	2/2	3/3
*trn*L intron	504	Undetermined	3	3/3	0/3	3/3
*trn*L intron	508	Undetermined	1	3/3	2/2	3/3
*trn*L intron	512	Undetermined	3	3/3	3/3	3/3
*trn*L intron	522	Undetermined	1	3/3	2/2	3/3
*trn*L intron	560	Undetermined	1, 3	6/6	5/5	6/6
*trn*L intron	584	Undetermined	3	0/3	0/3	3/3
*trn*L intron	604	Undetermined	3	3/3	3/3	3/3
*trn*L intron	696	*Populus tremuloides*	n/a	0/6	5/5	6/6
*trn*L-*trn*F intergenic spacer	179	Undetermined	5	3/3	3/3	3/3
*trn*L-*trn*F intergenic spacer	300	Undetermined	4	3/3	3/3	3/3
*trn*L-*trn*F intergenic spacer	324	Undetermined	4	3/3	3/3	3/3
*trn*L-*trn*F intergenic spacer	387	Undetermined	4, 5	6/6	6/6	6/6
*trn*L-*trn*F intergenic spacer	391	Undetermined	4	3/3	3/3	3/3
*trn*L-*trn*F intergenic spacer	394	*Bromus inermis* ^b^	n/a	3/3	6/6	9/9
*trn*L-*trn*F intergenic spacer	402	Undetermined	4	3/3	3/3	3/3
*trn*L-*trn*F intergenic spacer	432	Undetermined	4	3/3	3/3	3/3
*trn*L-*trn*F intergenic spacer	443	*Bromus inermis* ^b^	n/a	3/3	6/6	9/9
*trn*L-*trn*F intergenic spacer	460	Undetermined	4, 5	6/6	3/3	6/6

DNA was extracted from leaves of an identified species and mixed in varied proportions with DNA extracted from unidentified roots in soil. Numerator is number of successful detections; denominator is number of trials. See text for description of soil source.

^a^Roots from individual soil samples yielded fragments of different sizes; the genomic material yielding these fragments occurred in an unknown proportion, which comprised the DNA template.

^b^This species yielded fragments of different sizes.

Regarding species pools assembled with DNA extracted from roots of unidentified species spiked with DNA extracted from leaves of known species, for the *trn*T-*trn*L intergenic spacer, no fragments were detected at the lowest concentration, 4.5 ng μL^−1^ (Table 3). Increasing the concentration of DNA to 45.5 ng μL^−1^, resulted in nearly 100% successful detections. For the *trn*L intron, at DNA concentrations of 0.9 ng μL^−1^, detections of fragments were 100% successful (Table 3). Detection success was unaffected by the concentration gradient for most fragments of the *trn*L intron, though some fragments disappeared with a decrease in DNA concentration. Detections were 100% successful for *trn*L-*trn*F intergenic spacer, however it is possible that there were species present in the mixed root samples that went undetected across all trials.

When data were pooled across all created communities for *trn*T-*trn*L intergenic spacer, the logistic regression model was statistically significant [χ^2^(3) = 60.73, P < 0.001, R^2^ = 0.50], and both fragment size (P = 0.022) and its interaction with DNA concentration (P = 0.012) influenced detection success. At low concentrations of DNA, smaller fragments were more likely to be detected than larger fragments. When data were pooled across all created communities for the *trn*L intron chloroplast region, the logistic regression model was statistically significant [χ^2^(3) = 43.8, P < 0.001, R^2^ = 0.40]; fragment size (P = 0.001) alone affected detection success. The likelihood of detection was higher for small than large fragments. Due to the relative homogeneity in detection success, we did not perform a logistic regression on data pooled for *trn*L-*trn*F intergenic spacer.

## Discussion

To identify methodological sources of discrepancies between plant species occurring above and belowground (false-negatives), we investigated the effects of species, DNA template concentration and fragment size on biases associated with amplification, in addition to determining the effects of sample storage, and species differences in extraction of DNA. Below we discuss how each step of the work stream may affect species detections using FAFLPs (Table 4), and provide recommendations.

**Table 4:** Sources and outcomes of potential biases in the use of fluorescent amplified fragment length polymorphisms

Stage of work stream	Potential source of bias	Outcome
DNA extraction	Tissue	DNA yield is higher for leaves than roots
	Species	DNA yield differs across species
	Storage	A single freeze–thaw reduces DNA yield
DNA amplification	Species	Some species amplify better than others. Their amplification efficiency also depends on the target region
	DNA concentration	There is a weak positive relationship between DNA concentration and amplification efficiency. Some fragments are not detected at low DNA concentrations; detection thresholds depend on target region
	Fragment size	Depending on target region, detection of smaller fragments tends to be more successful than for large fragments (>600 bp)

### Biases with DNA extraction

We found evidence for several possible causes of false-negatives at the extraction step, however none stand out as being particularly troublesome. First, storage of samples affected DNA yield, specifically, freezing and thawing reduced yield. Sample preservation is well known to affect total concentration of extracted DNA [18 and references therein]. In addition to preserving DNA quality and quantity, field location, transport type and duration, number of samples, and size of samples will also affect choice of storage. Preserving soil samples rather than immediate processing of fresh material collected from the field is common practice, and would be difficult to circumvent in field studies. While avoiding bias among samples is relatively easy to control—similar storage techniques should be employed for all samples under consideration—storage itself may affect the quantity and quality of DNA to the extent that false-negatives ensue. Methods of extraction may also influence DNA yields. For instance, DNA yields from roots of *Bromus inermis* in our study were an order of magnitude higher than values reported by Bainard et al. [18] (220 versus 20 ng mg^−1^ dry tissue). This discrepancy is most likely due to the choice of kit used in extractions; we used PowerSoil^®^ DNA Isolation Kits (MO BIO Laboratories, Inc., Carlsbad, CA, USA) designed to process samples from ‘difficult’ soils, and they used DNeasy Plant Mini Kit (Qiagen Inc, Mississauga, ON, Canada). Following thawing, if DNA yield is below the amount needed for amplification, ethanol precipitation (see “Methods”) may be helpful in increasing DNA concentrations. In our study, that the A_260_/A_280_ absorbance ratios were on average 1.8 and unaffected by sample condition indicates that DNA purity is not affected as much as DNA quantity with thawing. However, degradation of DNA (breaking up of DNA into smaller fragments), when it occurs in roots, may be an unresolvable issue resulting in false-negatives.

**Figure 3:**
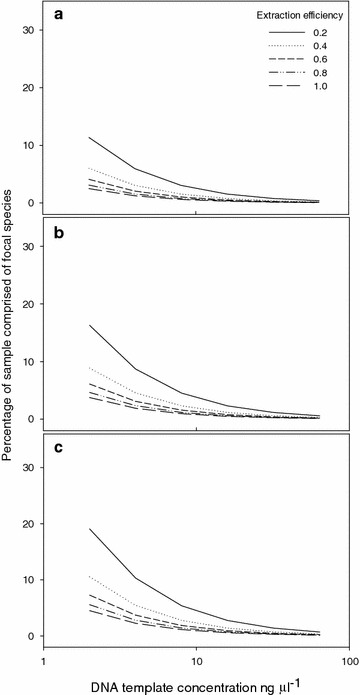
Relationship between DNA template concentration, extraction efficiency and detection thresholds. Shown is the extraction efficiency of a target species compared with matrix species, and the detectable percentage of sample comprising the target species across a range of the minimum DNA concentration required for amplification: **a** 0.05, **b** 0.075 and **c** 0.09 ng μL^−1^. Let D_s_ be the percentage of root material of the target species in the mixture, then the portion of the target species in the DNA extract, P_s_ = D_s_ × E_s_/(D_s_ × E_s_ + (100 − D_s_) × E_m_), where E_s_ is the DNA extraction efficiency of the target species, in ng μL^−1^ mg^−1^, and E_m_ is the DNA extraction efficiency of the other species in the matrix, in ng μL^−1^ mg^−1^. If C_smin_ is the minimum DNA concentration of the target species required for successful amplification of the focal fragment, in ng μL^−1^, then the minimum concentration of the DNA template required for amplification, C in ng μL^−1^, is given by C = C_smin_/P_s_, where C = C_smin_ × (D_s_ × E_s_ + (100 − D_s_) × E_m_)/(D_s_ × E_s_) or C = C_smin_ × (1 + ((100 − D_s_)/D_s_) × (E_m_/E_s_)).

Second, species differed in the amount of DNA yielded from extractions. Specifically, DNA yield was higher for *Bromus inermis* than *Populus tremuloides*. DNA yields may differ between species due to differences in gene composition, genome size, the presence of inhibitory substances (e.g. phenolics and polysaccharides), age of tissue, size of roots, and number of cells present in tissue. Ours is not the first report of variation among species in DNA yield from roots [e.g. 13, 16, 20]. In studies on mixed root samples, discrepancies between above and belowground species detections may be in part due to differences in extraction efficiency between species. For instance, we found that the DNA yield of *Populus tremuloides* was 74 ng mg^−1^ (mean from roots and shoots combined), approximately 40% that of *Bromus inermis*. This difference in extraction efficiency between the two species effectively increases the amount of tissue material required to equalize DNA concentrations, and the effective increase compounds with amplification requirements (Figure 3). For DNA markers which require high template concentrations, i.e., the *trn*T-*trn*L intergenic spacer, interspecific differences in extraction efficiencies may give rise to higher rates of false-negatives than markers which require lower template concentrations. Template inhibition, where single-stranded template molecules hybridize with each other rather than binding with primers, likely sets an upper limit on the concentration of DNA template permitting amplification [23]. For instance, Fisk et al. [20] found that roots making up a small fraction of mass in mixtures had disproportionately higher sequence representation in clone libraries relative to those making up larger fractions. Species with an initially low concentration of DNA may be selected for amplification resulting in bias in the final product against the initially abundant species.

### Biases with DNA amplification

Another cause which might underlie false-negatives is that for a given DNA template concentration, amplicon yield differed across species (and thus fragment sizes), and the difference in yield varied by chloroplast region. This finding suggests that copy numbers of the cpDNA regions vary among species and/or fidelity between primers and DNA is inconsistent across the three regions. However, this result only underscores the restriction on the use of FAFLPs: this method should not be used for quantifying abundance of species rather it should solely be used for detecting their presence. Although species vary in the amplicon production with PCR, so long as the amplified fragments are present in quantities above detection limits of the capillary sequencer, presence of these species ought to be detected using FAFLPs.

Of the methodological factors tested, those which are likely biasing detection of fragments, is the presence of size-based fragment competition. For each of the chloroplast regions, we pooled data across the created communities which included DNA from roots and leaves of various species. As a consequence, the source and amount of DNA across trials was highly variable, not unlike what we would expect from mixed template derived from field samples. We found that at low concentrations of DNA, larger fragments of the *trn*T-*trn*L intergenic spacer were less likely to be detected than smaller fragments, the likelihood of detection was higher for smaller than larger fragments yielded from the *trn*L intron, and detection success was overall high for *trn*L-*trn*F intergenic spacer. There were, however, exceptions to these trends suggesting other factors may affect detection in addition to fragment length. Other sources of amplification bias in community analysis include sequence composition (e.g. GC content) [24], interference from DNA flanking the template region [25], and the type of polymerase used [26].

We found it difficult to extract DNA in sufficient quantity to successfully amplify the *trn*T-*trn*L intergenic spacer. Notably, amplifications for three of eight species (Table 1) were unsuccessful in our study, matching rates reported by Taggart et al. [10]. In other reports, amplification of the *trn*T-*trn*L intergenic spacer has been met with less success than the *trn*L intron and *trn*L-*trn*F intergenic spacer [8, 10, 27]. For these reasons, in addition to evidence suggesting size-based fragment competition with amplification, we do not recommend use of this region for FAFLPs until further improvements can be made to improve extraction yields, which may also alleviate fragment competition. Studies often report this region contains greater sequence variation than the other two regions used in our study [28], thus it would be of interest to improve PCR-amplification of this region.

Larger fragments of the *trn*L intron were less likely to be detected than smaller fragments regardless of template concentrations. This result is driven by the unsuccessful detection of two species, *Chamerion angustifolium* and *Populus tremuloides*, which also yield the largest fragment sizes tested for this region, 603 and 706 bp, respectively (Table 3). This result reveals a difficulty in tests for size-based fragment competition, that is, fragment size is confounded with species identity. DNA extracted from some species is notoriously difficult to amplify [29]. As such, the unsuccessful detection of these two species begs the question whether it was due to size-based fragment competition or species-specific traits inhibiting amplification? For instance, Taggart et al. [10] stated that the rate of false-negatives increases with the number of ‘difficult’ species added to a mixture. The species identified as difficult in their study had fragment sizes of the *trn*L intron greater than 616 bp long. In our study, when amplified in isolation of other species both *Populus tremuloides* and *Chamerion angustifolium* were detected successfully, however, when amplified in mixture, they were not. Examining results between the studies, we suggest rather than species-specific traits inhibiting amplification, it is selection against fragment sizes approximately >600 bp that underlies these outcomes. The best test of size-based fragment competition on detection success would include fragments differing in size but present in similar quantities from a single species compared with those from several species to unequivocally rule out species-specific influences on amplification. Of course, it is difficult to create such a test because the majority of plants produce fragments of a single size for each chloroplast region [8, 10]. Why size-based fragment competition occurred in mixtures of fragments amplified from the *trn*T-*trn*L intergenic spacer and the *trn*L intron, but not the *trn*L-*trn*F intergenic spacer is unclear.

### Other origins of false-negatives and false-positives: intraspecific variation and the importance of the chosen species pool

Most of the species used in our study showed intraspecific variation, i.e., there was a continuous range of fragment sizes which emerged across individuals. Some species for example, *Bromus inermis* and *Picea glauca*, yielded two fragments of discrete sizes from single individuals. The presence of intraspecific variation highlights several essentials when employing the use of FAFLPs—to ignore these procedures may affect rates of false-negatives and false-positives. First, as is widely established, users must sample multiple individuals of a single species when building reference keys for assigning species identities to fragments produced by FAFLPs. Second, users must rely on multiple DNA markers to resolve species identities; for example, overlap in fragment sizes as observed in the *trn*L-*trn*F intergenic spacer precludes its use in isolation of other markers for determining species identities of roots. In another study, Randall et al. [8] were unable to differentiate between two species of *Picea* based on fragment lengths of the *trn*T-*trn*L intergenic spacer, the *trn*L intron, and the *trn*L-*trn*F intergenic spacer. Testing other non-coding regions of plastids for high variability will have applications in systematics, evolutionary biology and plant community ecology, where the use of molecular barcoding has recently been put into use [28]. The presence of fragments of discrete sizes produced by a single individual is indicative of either polymorphisms within a particular chloroplast region, the presence of multiple primer binding sites within a genome, or primer infidelity. We ruled out primer infidelity as both fragments produced by DNA extractions from roots occurred in equal abundance. We cannot discern between polymorphisms or multiple primer binding sites underlying the presence of multiple fragments; however, the latter is possible. For all primers aside from “F” used in our study, multiple binding sites exist across the genome of *Brachypodium distachyon* (L.) P. Beauv., a model monocot. Specifically, we found three binding sites of primer A, three of primer B, three of primer C, four of primer D, four of primer E and one of primer F. Regardless of its cause, the presence of multiple fragments increases the number of identifiers for a given species, however it may also increase the rate of false-positives. This result underscores the necessity of developing a reference key for each plant community of interest.

## Conclusions

In this study, we highlighted some methodological issues affecting false-negatives in the species identification of roots using FAFLPs. In particular, we focused on the consequences of uneven root abundances of co-occurring species and the presence of sized-based fragment competition during amplification. We do not recommend use of the *trn*T-*trn*L intergenic spacer for FAFLPs until further improvements can be made to improve extraction yields, which may alleviate size-based fragment competition. Size-based fragment competition was detected in FAFLPs of both the *trn*L intron and the *trn*L-*trn*F intergenic spacer. For any DNA-marker, we recommend users check for the disproportionate absence of species detected belowground versus aboveground as a function of fragment size. Detection of fragments yielded from amplification of the *trn*L-*trn*F intergenic spacer was the most successful, indicating its reliability; however this region should not be used alone in light of increased rates of false-positives with reliance on any single region.

## Methods

### DNA extraction: testing for differences in DNA yield and purity by tissue, species and sample condition

Appropriate permission was obtained for all collections of plant material used in this study. To test factors affecting DNA yield and purity, comparisons were made between and among tissues of a tree (*Populus tremuloides* Michx.) and a grass (*Bromus inermis* Leyss.). Invasion by *Bromus inermis* has been reported in both grasslands and disturbed forests [30–33], of which *Populus tremuloides* can be a dominant species. Approximately 20 g of leaves and roots were separately sampled for each of *Populus tremuloides* and *Bromus inermis*. Samples were collected from six *Populus tremuloides* plants, three of which were germinated from seeds collected from trees growing in Edmonton, Alberta, Canada and grown in the University of Alberta (UAlberta) greenhouse for 16 weeks. The other three samples were from mature trees occurring near the UAlberta campus, each separated by approximately 300 m. Leaf and root samples of *Bromus inermis* were located from six plants separated by approximately 300 m growing on the north perimeter of UAlberta campus. For field collections of both *Populus tremuloides* and *Bromus inermis*, care was taken to ensure root systems were attached to the focal plant.

Tissue samples were subjected to different numbers of freeze–thaw cycles: none (i.e., samples were fresh), one, two or three. Freeze–thaw cycles involved freezing samples at −20°C for 3 days, then thawing to room temperature for 2–3 h. After samples were subjected to the different levels of freeze–thaw cycles, they were lyophilized. Prior to this step, adhering debris was removed from roots by washing thoroughly under a gentle stream of tap water, followed by a rinse with deionized water and air-drying for 20 min. Samples were placed into perforated tin foil packets and lyophilized using a benchtop freeze dryer (Labconco FreeZone 2.5, Kansas City, MO, USA) for three days. Once dried, plant tissue was ground using a TissueLyser II (Qiagen Inc, Mississauga, ON, Canada). Fragmented tissue was placed in a 20 mL stainless steel milling jar with a 20 mm diameter grinding ball, and shaken at 30 Hz for 30 s. Twenty milligrams of ground leaf tissue was used to extract total cellular DNA using a DNeasy Plant Mini Kit (Qiagen Inc, Mississauga, ON, Canada) following the manufacturer’s directions. Leaf DNA was eluted in 50 μL of Buffer AE. We used PowerSoil^®^ DNA Isolation Kits (MO BIO Laboratories, Inc., Carlsbad, CA, USA) to extract genomic DNA from 15 to 50 mg of ground root tissue following the manufacturer’s directions. Root DNA was eluted in 50 μL of elution buffer. Both leaf and root DNA extracts were then further purified by ethanol precipitation. In a 1.5 mL micro-centrifuge tube, 20 μL of DNA extract was mixed with 2 μL of NaOAc–EDTA buffer (3 M sodium acetate with 125 mM ethylenediaminetetraacetic acid in water, pH 8.0), followed by the addition of 50 μL of ice-cold 95% ethanol and gently vortexed. The solution was kept at 4°C for 15 min, and then centrifuged at 10,000*g* for 15 min at 4°C. Supernatant was then aspirated and 70 μL of ice-cold 70% ethanol was added to the remaining DNA pellet and gently vortexed. The tube was again centrifuged at 10,000*g* for 5 min at 4°C and supernatant was aspirated. The purified DNA pellet was dried in a SpeedVac Concentrator (Savant Instruments, Inc., Farmingdale, NY, USA) for fifteen minutes, and then reconstituted in 20 μL of water. DNA yield and purity were quantified using a Nanodrop 2000 (Thermo Fisher Scientific, Wilmington, DE, USA). Purity of the extracted DNA was based on the ratio of absorbance at 260 and 280 nm, with pure DNA having a ratio between 1.8 and 2.0. Extracts were subsequently stored at −20°C for downstream activities.

### Amplification: testing the effect of species and DNA concentration on amplicon yield

Using the same purified DNA extract from freshly collected leaf samples of *Populus tremuloides* and *Bromus inermis*, we tested whether fragment yield of three cpDNA regions varied by DNA template concentration and species. We based our test on three individuals of each species, and added three individuals of a third species, *Melilotus officinalis* (L.) Pall., collected near Fort McMurray, Alberta, Canada, from which DNA had been extracted a year earlier using methods described above, and stored at −20°C. We manipulated DNA template concentrations by first bringing all the DNA extracts to their highest concentration possible via ethanol precipitation. In a separate test, we found that the three regions varied in the optimal concentration of DNA required for amplification. Specifically, amplifications were successful using 20–290 ng μL^−1^, 0.6–1.6 ng μL^−1^, and 0.6–1.6 ng μL^−1^ of DNA for the *trn*T-*trn*L intergenic spacer, *trn*L intron, and *trn*L-*trn*F intergenic spacer, respectively. Serial dilutions were made in steps of 0.5x, resulting in template concentrations of 512, 256, 128, 64, 32, and 16 ng μL^−1^for amplification of *trn*T-*trn*L intergenic spacer and 4, 2, 1, 0.5 and 0.25 ng μL^−1^ for amplification of the *trn*L intron and *trn*L-*trn*F intergenic spacer.

We amplified the three non-coding regions of chloroplast DNA using universal primers [34]: (1) the *trn*T-*trn*L intergenic spacer with primers A (5′-CATTACAAATGCGATGCTCT-3′) and B (5′-TCTACCGATTTCGCCATATC-3′), (2) the *trn*L intron with primers C (5′-CGAAATCGGTAGACGCTACG-3′) and D (5′-GGGGATAGAGGGACTTGAAC-3′) and (3) the *trn*L-*trn*F intergenic spacer with primers E (5′-GGTTCAAGTCCCTCTATCCC-3′) and F (5′-ATTTGAACTGGTGACACGAG-3′). Different coloured fluorescently labelled primers were used in PCR for each primer pair (primer A: FAM; primer C: VIC; primer E: NED; Integrated DNA Technologies, Coralville, Iowa, USA). Polymerase chain reactions were conducted in volumes totaling 25 μL: 12.5 μL of EconoTaq PLUS 2X Master Mix (Lucigen Corp., Middleton, WI, USA), 2.5 μL of each forward and reverse primer at 10 μM, 6.5 μL autoclaved deionized water, and 1 μL of 0.6–290 ng μL^−1^ DNA template. Amplifications were performed using an Eppendorf Mastercycler Pro S gradient thermal cycler (Model 6321; Eppendorf Canada, Mississauga, ON, Canada). Each region had unique thermal cycler conditions: (1) *trn*T-*trn*L intergenic spacer; 94°C for 5 min, 2 cycles of 94°C for 45 s, 56°C for 60 s, 72°C for 80 s, followed by 33 cycles of 94°C for 45 s, 61.5–0.3°C per cycle for 60 s, 72°C for 80 s and a final extension of 72°C for 30 min; (2) *trn*L intron; 94°C for 5 min, 2 cycles of 94°C for 60 s, 60°C for 60 s, 72°C for 80 s, followed by 33 cycles of 94°C for 60-s, 59.6–0.4°C per cycle for 60-s, 72°C for 80-s and a final extension of 72°C for 30 min; (3) *trn*L-*trn*F intergenic spacer, 94°C for 5 min, 2 cycles of 94°C for 60-s, 60°C for 60-s, 72°C for 80-s, followed by 33 cycles of 94°C for 60-s, 63–0.4°C per cycle for 60-s, 72°C for 80-s and a final extension of 72°C for 30 min [10]. Later in the study, it was found that the PCR conditions of *trn*L intron and *trn*L-*trn*F intergenic spacer could be used interchangeably with no reduction in amplification. Subsequently, the PCR conditions of *trn*L-*trn*F intergenic spacer were used for both regions for the rest of the study. Products were visualized by gel electrophoresis (1% agarose gel). Amplified products from the three loci were diluted 200× by combining 199 μL milliQ H_2_O and 1 μL of PCR product. From this dilution, 2 μL were added to 8 μL of HiDi formamide and 0.3 μL of GeneScan 1200 LIZ size standard (Applied Biosystems, Foster City, CA, USA). Note that by using differently colored fluorescently labelled primers, products across the three regions could be pooled; however, we chose to run the regions separately to increase the precision in fragment sizing. The final mixture was denatured at 94°C for 2 min and coldsnapped to maintain single-stranded fluorescently labelled DNA. Sizes of PCR amplicons were first resolved using capillary electrophoresis (ABI 3730 DNA analyzer; Applied Biosystems, Foster City, CA, USA) and then sized with GeneMapper (Applied Biosystems, Foster City, CA, USA) with GeneScan 1200 LIZ size standard. Fragment sizes read by the capillary sequencer were rounded to the nearest base pair. As part of this test, we confirmed that fragment sizes did not differ between roots and leaves, and by storage condition for both *Populus tremuloides* and *Bromus inermis* (n = 6). Amplicon yield was represented by the height of peaks (relative fluorescent units: rfu) detected by the capillary sequencer. Relative fluorescent units are the emission intensity of the fluorophores in samples registered as electrical voltage. This emission intensity is proportional to the molar concentration of the fluorophores, which is the same as the molar concentration of the amplicons since each fluorescently labelled amplicon carries one fluorophore.

### Amplification: testing the effect of DNA concentration and fragment size on fragment detection

To test whether size-based fragment competition and DNA concentration affects detection of amplified fragments, we created known communities containing DNA extracted from leaves of *Picea glauca* (Moench) Voss, *Melilotus officinalis*, *Sonchus arvensis* L., *Populus tremuloides*, *Bromus inermis*, *Chamerion angustifolium* (L.) Holub., *Trifolium hybridum* and *Rubus idaeus* L. These species are widespread across western Canada [35]. *Melilotusofficinalis*, *Sonchus arvensis* and *Bromus inermis* are typical invaders of boreal forests. The communities were constructed with the goal of capturing a range of fragment sizes for a given chloroplast region (see Table 1 for fragments sized in this study and previous unpublished trials). Though this test cannot discern whether underrepresentation of long fragments is due to either less efficient PCR of longer fragments during amplification, or bias in the electrophoresis of different fragment lengths during quantification, previous research shows the latter is unlikely to occur [8]. For the *trn*T-*trn*L intergenic spacer region, we mixed DNA extract of four species, *Picea glauca*, *Populus tremuloides*, *Bromus inermis* and *Melilotus officinalis*, in equal and unequal proportions replicated three times (Additional file 1: Table S1). We used a similar approach for the other two chloroplast regions using *Melilotus officinalis*, *Sonchus arvensis*, *Chamerion angustifolium* and *Populus tremuloides* for *trn*L intron, and *Trifolium hybridum*, *Chamerion angustifolium*, *Bromus inermis* and *Rubus idaeus* for *trn*L-*trn*F intergenic spacer. DNA template concentration was 50 ng μL^−1^for amplification of *trn*T-*trn*L intergenic spacer, and 1 ng μL^−1^for amplification of both the *trn*L intron and *trn*L-*trn*F intergenic spacer. Thus, we held the template concentration constant across trials, but varied the concentration of individual species comprising the DNA template (Additional file 1: Table S1). Sample preparation for *Populus tremuloides* and *Bromus inermis* is described above. Extractions of DNA of the remaining species were performed a year earlier using the same methods outlined above and stored at −20°C. These latter samples originated from single plants occurring in the Fort McMurray region, Alberta, Canada.

We also ran a similar trial on fragments yielded from roots with undetermined species identities spiked with DNA extracted from leaves of a known species (Additional file 1: Table S2). The roots had been sieved from 500 g soil samples collected from five locations in reclaimed boreal forest located in the Fort McMurray region of Alberta, Canada. Common understory species present on reclaimed areas included: *Chamerion angustifolium*, *Sonchus arvensis*, *Salix bebbiana* Sarg., *Melilotus officinalis*, *Trifolium hybridum*, and *Rubus idaeus* under a canopy of *Picea glauca* and *Populus tremuloides*. We extracted DNA from roots using methods described under ‘*DNA extraction:Testing for differences in DNA yield and purity by tissue, species and sample condition’*; these samples had been stored for 7 months at −20°C. In these trials, we did separate combinations of DNA extracted from roots of two of the five soils mixed with DNA extracted from leaves of a single known species (Additional file 1: Table S2). Thus, we held the template concentration constant across trials, but varied the concentration of known species comprising the DNA template, and in the case of roots, the concentration of mixed DNA template arising from the putative presence of multiple species (Additional file 1: Table S2).

### Data analysis

To test for differences in DNA yield and purity by tissue, species and sample condition, we used general linear models with species, tissue, sample condition, and their interactions as fixed explanatory effects. To predict fragment yield (relative fluorescent units), we used a linear mixed effects model with DNA concentration, species and individual (n = 3) as fixed factors, and individual nested within species as a random factor. Relative fluorescent units were summed across fragments differing in size. We analyzed combined data from created species mixtures (roots and leaves) for each chloroplast region separately using logistic regression to test the effects of fragment size, DNA concentration and their interaction on detection success. All analyses were performed in IBM SPSS Statistics for Windows, Version 21.0 (IBM Corp., Armonk, NY, USA).

## Additional file

Additional file 1: In the Supplemental Material Section details on the experimental assembly of plant communities, and results of all statistical tests are presented.

## Abbreviations

A: absorbance
bp: base pairs
cpDNA: chloroplast DNA
DNA: deoxyribonucleic acid
EDTA: ethylenediaminetetraacetic acid
FAFLPs: fluorescent amplified-fragment length polymorphisms
PCR: polymerase chain reaction
Rfu: relative fluorescent units
