# Supplementary material for: Biases underlying species detection using fluorescent amplified-fragment length polymorphisms yielded from roots
Source: Plant Methods. 2015 Jun 26;11:36. doi: 10.1186/s13007-015-0079-1 (PMC4480983; doi:10.1186/s13007-015-0079-1)
Supplement: Additional file 1: — In the Supplemental Material Section details on the experimental assembly of plant communities, and results of all statistical tests are presented. [file 13007_2015_79_MOESM1_ESM.docx]

**Appendix tables**

Table A1: DNA concentration (ng μL ^-1^) of species amplified alone or in combination with other plant species. For mixed species trials, three replicates were performed. Species abbreviations: Pic.gla: *Picea glauca*; Pop.tre: *Populus tremuloides*; Bro.ine: *Bromus inermis*; Mel.off: *Melilotus officinalis*; Son.arv: *Sonchus arvensis*; Cha.ang: *Chamerion angustifolium*; Tri.hyb: *Trifolium hybridum*; Rub.ide: *Rubus ideaus*.

|  | Species | | | | | | | |
| --- | --- | --- | --- | --- | --- | --- | --- | --- |
| Chloroplast region amplified | Pic.gla | Pop.tre | Bro.ine | Mel.off | Son.arv | Cha.ang | Tri.hyb | Rub.ide |
| *trn*T-*trn*L intergenic spacer | 50 | 0 | 0 | 0 | 0 | 0 | 0 | 0 |
| *trn*T-*trn*L intergenic spacer | 0 | 50 | 0 | 0 | 0 | 0 | 0 | 0 |
| *trn*T-*trn*L intergenic spacer | 0 | 0 | 50 | 0 | 0 | 0 | 0 | 0 |
| *trn*T-*trn*L intergenic spacer | 0 | 0 | 0 | 50 | 0 | 0 | 0 | 0 |
| *trn*T-*trn*L intergenic spacer | 12.5 | 12.5 | 12.5 | 12.5 | 0 | 0 | 0 | 0 |
| *trn*T-*trn*L intergenic spacer | 22.73 | 22.73 | 2.27 | 2.27 | 0 | 0 | 0 | 0 |
| *trn*T-*trn*L intergenic spacer | 2.27 | 2.27 | 22.73 | 22.73 | 0 | 0 | 0 | 0 |
| *trn*L intron | 0 | 0 | 0 | 1 | 0 | 0 | 0 | 0 |
| *trn*L intron | 0 | 0 | 0 | 0 | 1 | 0 | 0 | 0 |
| *trn*L intron | 0 | 0 | 0 | 0 | 0 | 1 | 0 | 0 |
| *trn*L intron | 0 | 1 | 0 | 0 | 0 | 0 | 0 | 0 |
| *trn*L intron | 0 | 0.25 | 0 | 0.25 | 0.25 | 0.25 | 0 | 0 |
| *trn*L intron | 0 | 0.05 | 0 | 0.45 | 0.45 | 0.05 | 0 | 0 |
| *trn*L intron | 0 | 0.45 | 0 | 0.05 | 0.05 | 0.45 | 0 | 0 |
| *trn*L-*trn*F intergenic spacer | 0 | 0 | 1 | 0 | 0 | 0 | 0 | 0 |
| *trn*L-*trn*F intergenic spacer | 0 | 0 | 0 | 0 | 0 | 1 | 0 | 0 |
| *trn*L-*trn*F intergenic spacer | 0 | 0 | 0 | 0 | 0 | 0 | 1 | 0 |
| *trn*L-*trn*F intergenic spacer | 0 | 0 | 0 | 0 | 0 | 0 | 0 | 1 |
| *trn*L-*trn*F intergenic spacer | 0 | 0 | 0.25 | 0 | 0 | 0.25 | 0.25 | 0.25 |
| *trn*L-*trn*F intergenic spacer | 0 | 0 | 0.05 | 0 | 0 | 0.45 | 0.45 | 0.05 |
| *trn*L-*trn*F intergenic spacer | 0 | 0 | 0.45 | 0 | 0 | 0.05 | 0.05 | 0.45 |

Table A2: DNA concentrations (ng μL ^-1^) of species amplified alone or in combination with DNA yielded from roots (mixed template) of undetermined species. For trials of mixed components (i.e., DNA from roots and leaves), three replicates were performed.

|  | Species of leaves | | | Origin of roots | | | | |
| --- | --- | --- | --- | --- | --- | --- | --- | --- |
| cpDNA amplified | *Populus tremuloides* | *Bromus inermis* | *Melilotus officinalis* | Soil A | Soil B | Soil C | Soil D | Soil E |
| *trn*T-*trn*L intergenic spacer | 0 | 0 | 50 | 0 | 0 | 0 | 0 | 0 |
| *trn*T-*trn*L intergenic spacer | 0 | 0 | 0 | 50 | 0 | 0 | 0 | 0 |
| *trn*T-*trn*L intergenic spacer | 0 | 0 | 0 | 0 | 50 | 0 | 0 | 0 |
| *trn*T-*trn*L intergenic spacer | 0 | 0 | 4.55 | 45.45 | 0 | 0 | 0 | 0 |
| *trn*T-*trn*L intergenic spacer | 0 | 0 | 45.45 | 4.55 | 0 | 0 | 0 | 0 |
| *trn*T-*trn*L intergenic spacer | 0 | 0 | 25 | 25 | 0 | 0 | 0 | 0 |
| *trn*T-*trn*L intergenic spacer | 0 | 0 | 4.55 | 0 | 45.45 | 0 | 0 | 0 |
| *trn*T-*trn*L intergenic spacer | 0 | 0 | 45.45 | 0 | 4.55 | 0 | 0 | 0 |
| *trn*T-*trn*L intergenic spacer | 0 | 0 | 25 | 0 | 25 | 0 | 0 | 0 |
| *trn*L intron | 1 | 0 | 0 | 0 | 0 | 0 | 0 | 0 |
| *trn*L intron | 0 | 0 | 0 | 1 | 0 | 0 | 0 | 0 |
| *trn*L intron | 0 | 0 | 0 | 0 | 0 | 1 | 0 | 0 |
| *trn*L intron | 0.09 | 0 | 0 | 0.91 | 0 | 0 | 0 | 0 |
| *trn*L intron | 0.91 | 0 | 0 | 0.09 | 0 | 0 | 0 | 0 |
| *trn*L intron | 0.5 | 0 | 0 | 0.5 | 0 | 0 | 0 | 0 |
| *trn*L intron | 0.09 | 0 | 0 | 0 | 0 | 0.91 | 0 | 0 |
| *trn*L intron | 0.91 | 0 | 0 | 0 | 0 | 0.09 | 0 | 0 |
| *trn*L intron | 0.5 | 0 | 0 | 0 | 0 | 0.5 | 0 | 0 |
| *trn*L-*trn*F intergenic spacer | 0 | 1 | 0 | 0 | 0 | 0 | 0 | 0 |
| *trn*L-*trn*F intergenic spacer | 0 | 0 | 0 | 0 | 0 | 0 | 1 | 0 |
| *trn*L-*trn*F intergenic spacer | 0 | 0 | 0 | 0 | 0 | 0 | 0 | 1 |
| *trn*L-*trn*F intergenic spacer | 0 | 0.09 | 0 | 0 | 0 | 0 | 0.91 | 0 |
| *trn*L-*trn*F intergenic spacer | 0 | 0.91 | 0 | 0 | 0 | 0 | 0.09 | 0 |
| *trn*L-*trn*F intergenic spacer | 0 | 0.5 | 0 | 0 | 0 | 0 | 0.5 | 0 |
| *trn*L-*trn*F intergenic spacer | 0 | 0.09 | 0 | 0 | 0 | 0 | 0 | 0.91 |
| *trn*L-*trn*F intergenic spacer | 0 | 0.91 | 0 | 0 | 0 | 0 | 0 | 0.09 |
| *trn*L-*trn*F intergenic spacer | 0 | 0.5 | 0 | 0 | 0 | 0 | 0 | 0.5 |

Table A3: General linear model testing influence of species (*Populus tremuloides* and *Bromus inermis*), tissue (roots and leaves) and sample condition (fresh, one, two and three freeze-thaw cycles) on DNA yield.

| Source | df (num,den) | F | P |
| --- | --- | --- | --- |
| Species | 1, 78 | 92.9 | <0.001 |
| Tissue | 1, 78 | 40.4 | <0.001 |
| Sample condition | 3, 78 | 7.98 | <0.001 |
| Species × Tissue | 1, 78 | 0.08 | 0.79 |
| Species × Sample condition | 3, 78 | 2.07 | 0.11 |
| Tissue × Sample condition | 3, 78 | 2.19 | 0.096 |
| Species × Tissue × Sample condition | 3, 78 | 2.42 | 0.073 |

Table A4: General linear model testing influence of species (*Populus tremuloides* and *Bromus inermis*), tissue (roots and leaves) and sample condition (fresh, one, two and three freeze-thaw cycles) on DNA purity (A_260_/A_280_ absorbance ratios).

| Source | df (num, den) | F | P |
| --- | --- | --- | --- |
| Species | 1, 78 | 1.38 | 0.24 |
| Tissue | 1, 78 | 15.92 | <0.001 |
| Sample condition | 3, 78 | 0.96 | 0.42 |
| Species × Tissue | 1, 78 | 6.29 | 0.014 |
| Species × Sample condition | 3, 78 | 1.42 | 0.24 |
| Tissue × Sample condition | 3, 78 | 0.29 | 0.83 |
| Species × Tissue × Sample condition | 3, 78 | 1.86 | 0.14 |

Table A5: Linear mixed effects model testing influence of species (*Populus tremuloides*, *Bromus inermis*, and *Melilotus officinalis*), DNA template concentration (DNA), and their interactions on fragment yield of the *trn*T-*trn*L intergenic spacer.

| Source | df (num, den) | F | P |
| --- | --- | --- | --- |
| Species | 2, 14.5 | 0.51 | 0.61 |
| DNA | 1, 30.9 | 17.9 | <0.001 |
| Species × DNA | 2, 31.3 | 7.5 | 0.002 |

Table A6: Linear mixed effects model testing influence of species (*Populus tremuloides*, *Bromus inermis*, and *Melilotus officinalis*), DNA template concentration (DNA), and their interactions on fragment yield of the *trn*L intron.

| Source | df (num,den) | F | P |
| --- | --- | --- | --- |
| Species | 2, 18.9 | 1015 | <0.001 |
| DNA | 1, 32.8 | 4.5 | 0.042 |
| Species × DNA | 2,32.8 | 2.1 | 0.15 |

Table A7: Linear mixed effects model testing influence of species (*Populus tremuloides*, *Bromus inermis*, and *Melilotus officinalis*), DNA template concentration (DNA), and their interactions on fragment yield of the *trn*L-*trn*F intergenic spacer.

| Source | df (num, den) | F | P |
| --- | --- | --- | --- |
| Species | 2, 11.8 | 6.6 | 0.012 |
| DNA | 1, 32.1 | 2.1 | 0.16 |
| Species × DNA | 2, 32.1 | 1.8 | 0.18 |
